# Inferior Outcome after Unstable Trochanteric Fracture Patterns Compared to Stable Fractures in the Elderly

**DOI:** 10.3390/jcm10020171

**Published:** 2021-01-06

**Authors:** Johannes Gleich, Carl Neuerburg, Christoph Linhart, Alexander Martin Keppler, Daniel Pfeufer, Christian Kammerlander, Wolfgang Böcker, Christian Ehrnthaller

**Affiliations:** Department of General, Trauma and Reconstructive Surgery, University Hospital, LMU Munich, 81377 Munich, Germany; johannes.gleich@med.uni-muenchen.de (J.G.); christoph.linhart@med.uni-muenchen.de (C.L.); alexander.keppler@med.uni-muenchen.de (A.M.K.); daniel.pfeufer@med.uni-muenchen.de (D.P.); christian.kammerlander@med.uni-muenchen.de (C.K.); wolfgang.boecker@med.uni-muenchen.de (W.B.); christian.ehrnthaller@med.uni-muenchen.de (C.E.)

**Keywords:** proximal femur fracture, trochanteric fracture, unstable, osteoporosis, orthogeriatric

## Abstract

Background: Various risk factors affecting outcome of elderly patients after proximal femur fracture have been identified. The present study aims to evaluate the impact of the fracture pattern in trochanteric fractures on postoperative mobility and complications. Methods: Ninety-two patients with a mean age of 84 years were included. According to the revised AO/OTA classification, fractures were divided into stable (AO 31A1) and unstable (AO 31A2/3) patterns. A follow-up examination was performed 12 months after cephalomedullary fixation to assess outcome parameters for mobility/activities of daily living (Parker Mobility Score (PMS)/Barthel Index (BI)) and complications (increase in requirement of care, hospital readmission, mortality rate). Results: At follow-up, patients with unstable trochanteric fracture patterns presented with lower PMS and BI compared to stable fractures (*p* < 0.05). Further, higher requirement of care and higher readmission rates compared to stable patterns were observed. Conclusion: Unstable trochanteric fractures presented inferior outcome compared to simple fracture patterns. This might be explained by the increasing surgical trauma in unstable fractures as well as by the mechanical impact of the lesser trochanter, which provides medial femoral support and is of functional relevance. Subsequent studies should assess if treatment strategies adapted to the specific fracture pattern (refixation of lesser trochanter) influence outcome in unstable trochanteric fractures.

## 1. Introduction

Suffering a proximal femur fracture is a life-changing event for elderly patients. A subsequent decline in activities of daily living and independency is common, and mortality rates up to 20% in the first year after fracture can be observed [1,2]. The annual amount of patients with proximal femur fractures, with growing incidence of trochanteric fractures, is expected to increase up to 6.3 millions per year by 2050 due to the aging population [3,4]. Therefore, better understanding of additional parameters influencing outcome after proximal femur fracture is essential. Besides preexisting comorbidities, prefracture mobility and independency, different fracture patterns appear to affect individual rehabilitation and outcome after trochanteric fractures.

The absolute goal for elderly patients after proximal femur fracture surgery is immediate full weight-bearing, as a significant increase in mortality was evaluated in patients treated with weight-bearing restrictions [5]. Gait analysis could show that patients with unstable trochanteric fractures had a reduction of weight-bearing on the affected leg compared to stable fracture patterns and, therefore, are at risk to realize these instructions [6]. Further, reduced weight-bearing after intramedullary fixation of trochanteric fractures compared to arthroplasty after femoral neck fractures was observed [7]. The categorization in stable and unstable fracture patterns was also applied in the revised AO/OTA classification of trochanteric fractures. Fractures with only two main fragments and an intact lesser trochanter, as well as fractures with an intact lateral wall (>20.50 mm) and a small lesser trochanter fragment may be considered stable after anatomical reduction and fixation (Type 31A1.1-3). Fractures with an insufficient lateral wall (≤20.50 mm) or with one or more intermediate fragments and subsequent posteromedial bone loss are unstable (Type 31A2.2-3.3) [8].

Besides type 31A3 fracture patterns, which are frequently treated by open reduction, cerclage-wiring and long intramedullary nailing, there is no relevant difference in the common treatment of type 31A1/2 trochanteric fractures, while fracture fixation via cephalomedullary nailing is of growing acceptance for the treatment of all trochanteric fracture patterns [9,10]. Following the current opinion no specific surgical treatment of the lesser trochanter fragment is necessary during intra- or extramedullary fixation of these fractures, although it is the insertion of the iliopsoas muscle, which plays an important role for hip flexion. Study findings are contrary: while refixation of the lesser trochanter was shown to increase biomechanical stability, functional disadvantages in patients without refixation as well as no effects on flexion power after untreated trochanter displacement were observed [11,12,13].

We hypothesized that the outcome of patients after intramedullary fixation of unstable trochanteric fracture patterns, regarding mobility, activities of daily living and upcoming complications, is reduced compared to patients presenting with stable fractures. 

## 2. Materials and Methods

This retrospective single-center study was approved and registered by the local ethics committee (Reg. No. 234-16). All patients aged ≥70 years admitted from 1 January 2014 to 31 December 2014 to a level one trauma center with a trochanteric fracture were included. AO/OTA type 31A1.1 fractures, conservative treatment and admission after refracture of the proximal femur were defined as the only exclusion criteria to give a realistic depiction on the common study population. All patients or their legal representative gave written informed consent for inclusion. The study was conducted in accordance with the Declaration of Helsinki.

**Figure 1:**
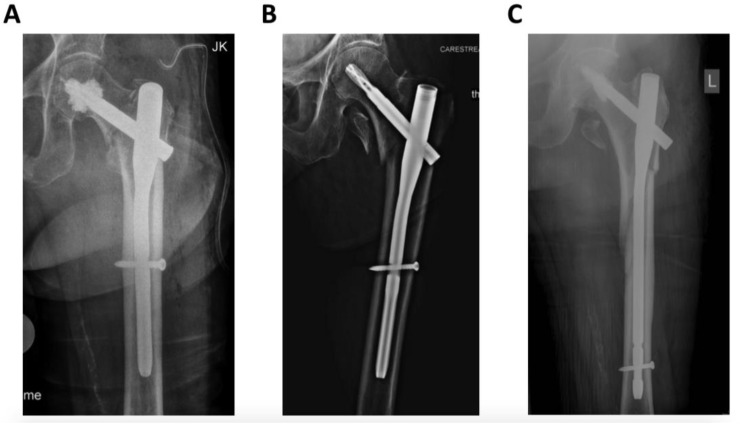
Radiographic images of different trochanteric fracture patterns according to the revised AO/OTA Classification. (**A**) = 31A1; (**B**) = 31A2; (**C**) = 31A3.

In all cases, surgery was performed under general anesthesia by trauma specialists (seven senior trauma surgeons), according to the AO principles of fracture management. An extension table was used for fracture reduction with open or closed reduction depending on the fracture pattern. Cephalomedullary fixation was achieved with the Proximal Femoral Nail Antirotation (PFNA, DePuy/Synthes, Umkirch, Germany) with additional bone cement augmentation of the helical blade in case of poor bone quality, according to the surgeons’ intraoperative findings (Figure 1). 

During their hospital stay, patients were treated by trauma surgeons and their team, and other medical specialties were involved on consultation basis. There was no implemented orthogeriatric service at the time of the study. Patients received one 30-min session of physiotherapy per day, starting on the first postoperative day. For all patients, mobilization was allowed without weight-bearing restrictions, using walking aids of choice if needed. Social workers were involved in the treatment shortly after surgery to plan subsequent rehabilitation and additional help needed after discharge (nursing service, walking aids).

Mobility was defined as the primary outcome, assessed with the Parker Mobility Score (PMS; scale 0–9, higher score presenting higher mobility indoors/outdoors and during shopping), which is a valid score for mobility assessment in hip fracture patients [14,15]. Secondary outcome parameters were activities of daily living (ADL) measured with the Barthel Index (BI) and occurring complications (assessed by changes in requirement of care (RC), readmission rate and mortality rates) [16]. 

Barthel Index was chosen for assessment of activities of daily living, as it is a reliable tool to evaluate the recovery of prefracture physical condition in hip fracture patients (range from 0 to 100, higher score suggests higher independence in daily living) [17]. 

According to the German health-care assurance, requirement of care is determined as the total amount of time a patient needs professional help in his activities of daily living and is assessed by specially qualified physicians of the insurance companies. RC was formerly specified as a degree from 1 to 3, which was extended up to five degrees in January 2017. The degree before hospital admission and at follow-up was requested.

Readmission rate was evaluated by retrospective analysis of the data management system of the study center and queried by the patients themselves (to register also readmission to another hospital). Reasons for readmission were separated into complications associated with initial surgery (wound healing disorder, implant failure), refracture (secondary fracture somewhere else) and other medical complications (myocardial infarction, pulmonary embolism, pneumonia, urinary tract infection). 

For follow-up, 12 months after their stay in hospital, each patient was contacted by phone and was sent a specifically designed questionnaire to assess the parameters mentioned above. If the patient could not answer by themselves, their next of kin or legal representative were asked for consent and information. In case no information was available via contact of the patients themselves, relatives, general practitioners, nursing homes or the local authorities, the patient was regarded as “lost to follow-up.” Fracture assessment was performed by two independent investigators (senior trauma surgeons) following the revised AO/OTA classification. Based on this assessment, patients were divided into three groups (I: stable patterns, AO 31A1.2/3; II: unstable trochanteric patterns AO 31A2.2/3; III: unstable subtrochanteric patterns AO 31A3.1-3) [8].

Baseline data were collected from the medical records and stored with a standardized data management file (Excel 2011, Microsoft Cooperation, Redmond, WA, USA). IBM SPSS Statistics, Version 26 (IBM Corp. Released 2016. Amonk, NY, USA) was used for statistical analysis. Data are reported as either mean +/− standard error of mean (SEM) or for categorical data as absolute frequency with a percentage distribution. After ruling out normal distribution using the Kolmogorov-Smirnov test, a Mann-Whitney-U-Test or *t*-test was used, while Fisher’s Exact Test was used for dichotome variables; for correlation analysis the Pearson correlation coefficient was used. A *p*-value < 0.05 was regarded to be statistically significant.

## 3. Results

### 3.1. Patient Characteristics

Ninety-two patients with a mean age of 84 ± 7.00 years were included (the initial study sample consisted of 122 patients; 30 patients were excluded due to an age < 70 years). Comparison of the baseline data revealed no major differences between the subgroups (Table 1). Age, sex and duration of inpatient treatment were equally distributed. Regarding comorbidities, the Charlson Comorbidity Index and American Society of Anesthesiologists (ASA) physical status classification showed no significant differences between the study groups. Most patients were admitted from their home and the majority needed help from nursing staff to manage their daily activities. Thirteen percent (*n* = 12) of the patients were lost to follow-up after 12 months with no significant difference in baseline data compared to the overall collective.

### 3.2. Surgical Treatment

**Figure 2:**
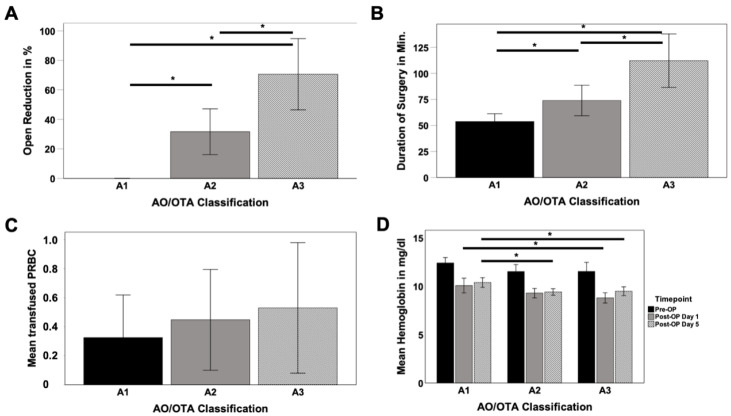
Requirement for open reduction (**A**), duration of the surgery (**B**), amount of perioperative transfused packed red blood concentrates (PRBC) (**C**) and impact in pre-/peri- and postoperative blood loss (**D**) depending on the fracture pattern; * *p* < 0.05.

Due to the different treatment regimes, frequency of open reduction significantly increased from stable 31A1 (0%) towards unstable 31A2 (32%) and 31A3 (71%) fractures (A1/A2 *p* < 0.001; A1/A3 *p* < 0.001; A2/A3 *p* = 0.01) as well as duration of the surgery (A1 = 54.00 min, A2 = 74.00 min, A3 = 112.00 min) (A1/A2 *p* = 0.016; A1/A3 *p* < 0.001; A2/A3 *p* = 0.01) (Figure 2A,B). While preoperative hemoglobin values did not differ, significant differences between 31A1 and 31A2 on postoperative day 5 (10%, *p* = 0.02) as well as between 31A1 and 31A3 for postoperative day 1 (13%; *p* = 0.007) and 5 (7%; *p* = 0.008) were observed (Figure 2D).

### 3.3. Mobility and Activities of Daily Living

**Figure 3:**
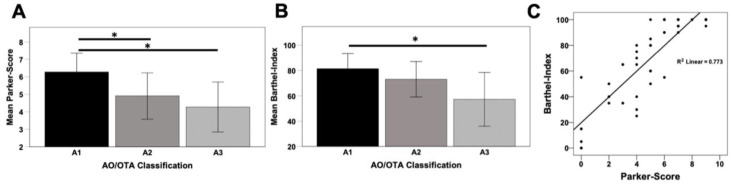
Mean values of Parker Mobility Score (**A**) and Barthel Index (**B**) in respect to the AO/OTA classification at 12 months follow-up and correlation of both parameters (**C**); * *p* < 0.05.

Functional outcome after 12 months was assessed by the Parker Mobility Score and the Barthel Index, which both presented significant differences depending on the fracture pattern (PMS: A1/A2 *p* = 0.035, A1/A3 *p* = 0.041; BI: A1/A3 *p* = 0.032). While patients with stable fracture patterns showed the highest values for PMS (A1 = 6.30; A2 = 4.70) and BI (A1 = 81.00; A2 = 73.00) at follow-up, this rate gradually decreased towards unstable 31A3 fractures (PMS = 4.30; BI = 57.00) (Figure 3A,B). PMS and BI also showed a significant correlation (R^2^ = 0.773; *p* < 0.001) (Figure 3C).

### 3.4. Complications

**Figure 4:**
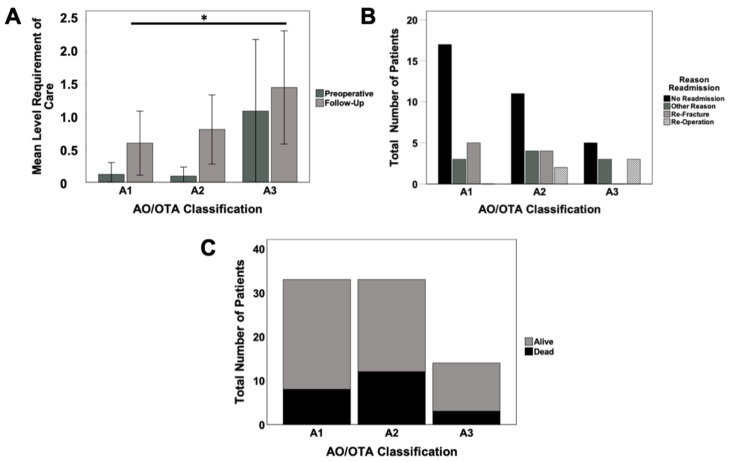
Requirement of care at preoperative and follow-up assessment (**A**), reasons for readmission (**B**) and mortality (**C**) in respect to the AO/OTA classification; * *p* < 0.05.

All study groups demonstrated an increase in requirement of care at follow-up assessment. While, for simple 31A1 fractures, the rate increased 5-fold from a mean of 0.12 to 0.60, unstable 31A2 fractures increased 8-fold from a mean of 0.10 to 0.80, the rate for unstable 31A3 fractures increased only 24% from a mean of 1.10 to 1.50. The difference between A1 and A3 fractures reached significance with *p* = 0.038 (Figure 4A).

Major differences were evaluated regarding the readmission rate between study groups. While in stable fracture patterns (31A1), only 32% of patients were readmitted, this rate increased towards 48% (31A2) and 55% (31A3) in unstable fractures. Reoperation rates as well as other reasons for readmission gradually increased towards unstable fracture patterns (A1 = 0%, A2 = 10%; A3 = 27%), while the rate of refractures was the same for patients in group I as well as in the more unstable fracture pattern group II. The highest rate for readmission for other medical reasons was seen in group II with 19% (Figure 4B). There was no significant difference in mortality rates between the fracture patterns (Figure 4C).

## 4. Discussion

In the present study, we hypothesized that the functional outcome in patients with unstable trochanteric fractures is reduced compared to patients presenting with stable fracture patterns. While there was no difference in baseline characteristics and time of hospitalization was comparable, functional abilities in the follow-up evaluation after 12 months and occurring complications showed significant differences. 

The Parker Mobility Score as well as the Barthel Index showed a growing decline from stable to unstable fracture patterns at the one-year follow-up evaluation. One possible reason might be the higher surgical trauma due to an increasing amount of open reduction, which was necessary in some 31A2 and especially 31A3 fractures. The duration of the surgery likewise increased significantly in unstable patterns. This might also have influenced blood loss, which was indirectly monitored by measurement of peri- and postoperative hemoglobin values as well as the amount of transfused PRBC and which indicated increasing blood loss for unstable fracture patterns. Therefore, the extent of the surgical approach may be a reason for functional impairment in unstable trochanteric fracture patterns; Zhang et al. evaluated that higher intraoperative blood loss results in lower survival in a geriatric hip fracture collective [18]. The increase in requirement of care after unstable trochanteric fractures compared to simple 31A1 fractures points out the decline of independence in activities of daily living and mobility, too. There was a high one-year mortality rate over 20% in all subgroups with no inter-group differences, which is consistent with current study findings [2].

During follow-up, a significantly higher readmission rate was demonstrated in patients with unstable fracture patterns. While only 27% of patients with simple trochanteric fractures (31A1) had to be admitted to a hospital again, this rate increased towards 50% in 31A2 fractures and 54% in 31A3 fractures. Having a closer look at the reason for rehospitalization, it becomes evident that surgical complications of the initial fracture treatments increased with instability of the fracture. While no patient in the simple, stable fracture group was readmitted due to surgical complications, this rate increased towards 8% in 31A2 fractures and up to 27% in 31A3 fractures. While it is not surprising that 31A3 fractures display more surgical complications due to their different treatment regime with open reduction and higher surgical trauma, the reason for differences between the other study groups remain unclear. Literature dealing with this question is scarce, but there is evidence for a worse outcome of intertrochanteric fractures compared to femoral neck fractures especially in the early postoperative course making the increase of instability the most likely reason for higher complication rates and reduced outcome at last [19,20]. 

The role of the lesser trochanter fragment remains unclear: a biomechanical study highlighted the importance for posteromedial stability in trochanteric fractures. Here, a refixation of the lesser trochanter fragment showed significantly increased stability and reduced medial dislocation of the femoral neck, which would finally lead to cut-out of the femoral neck screw [11]. A simultaneous clinical study reported improved functional outcome according to the Harris Hip Score after double loop cerclages compared to single loop cerclages for additional fracture reduction following nailing of trochanteric fractures, yet the study group was rather small with a limited follow-up period [21]. Contrary to these findings, Schenkel et al. demonstrated no significant differences in flexion force of the hip after fracture fixation of trochanteric fractures with unfixed lesser trochanter fragments; only fatty infiltration of the iliopsoas muscle was observed [13]. 

Micromovement within a more complex fracture pattern following fracture fixation might be causative for a reduced mobilization in these patients, which subsequently leads to differences in functional outcome as observed between the different groups. It is well known that muscle disuse caused by reduced mobilization is associated with accelerated loss of muscle strength, which is difficult to recover from. Furthermore, deficits in physical function predict short term falls in older adults, which might provoke further fractures and immobilization [22]. Even a long-term experience of pain following fragility fractures has been reported by Gheorghita et al. [23]. Therefore, there is an ongoing discussion about the most suitable rehabilitation protocol after cephalomedullary fixation of unstable trochanteric fractures with observed advantages and disadvantages for weight-bearing restrictions as well as full weight-bearing recommendations [24]. Regardless of this general discussion, it has been shown that elderly patients are struggeling to maintain postoperative weight-bearing restrictions after proximal femur fracture, while any of these aftercare recommendations can trigger long-term immobilization [25]. 

The missing posteromedial support could have led to the higher surgical complication rate in the present study collective, subsequently leading to loss of reduction and readmission. Restoration of the posteromedial cortical continuity was already stated in 1948 as key to a stable fracture reduction [26]. Although implant techniques have improved dramatically ever since, Knobe et al. performed a meta-analysis in which they found that the absence of the medial support was considered the main criterion for fracture instability (84%), whereas a broken lateral wall and detached greater trochanter were considered by 5% of the respondents to determine instability [22]. The authors also showed that only two percent of the queried surgeons routinely fixed unstable trochanteric fractures with extramedullary devices [22]. 

Some limitations have to be taken into account: even if a large collective of patients suffering different trochanteric fracture patterns was analyzed and significant differences were observed between the groups, the amount of patients in the subgroups is limited. Due to the retrospective study design, there was no evaluation of the Barthel Index (BI) prior to surgery. Therefore, no information could be obtained if some patients had lower scores before injury, yet there is valid data showing that the BI steadily increases in the first year after proximal femur fracture, which strengthens the usability of the BI for analysis in the present study [17]. Potential differences in muscle mass, which might have affected the observed differences in outcome between the groups, also need to be taken into account. There was no assessment of subjective parameters influencing mobility like the Visual Analog Scale for Pain (VAS Pain). As all patients received standardized pain medication following WHO (World Health Organization) treatment guidelines, which were adjusted individually to allow painless walking, this might have only little influence on overall outcome, but should be assessed in subsequent studies.

## 5. Conclusions

The data of the present study on patients with different trochanteric fracture patterns clearly indicates that proximal femur fractures remain a life-changing event. In most studies, trochanteric fractures are subsumed into one collective. This study highlights the fact that different fracture patterns should be respected with regards to their stability and their possible complications, expected functional outcome and postoperative rehabilitation process. Further clinical studies are needed to clarify if increased stability, e.g., by refixation of the lesser trochanter, could increase functional outcome in unstable 31A2 and 31A3 fracture patterns.

## Figures and Tables

**Table 1:** Patient demographics and baseline data.

	Group I (A1.2/3) *n* = 37	Group II (A2.2/3) *n* = 38	Group III (A3.1-3) *n* = 17	*p*-Value
**Age**	83 ± 6.40	85 ± 6.70	84 ± 7.80	ns
**Gender**				ns
Female	76%	82%	80%	
Male	24%	18%	20%	
**ASA**				ns
1	0%	3%	0%	
2	43%	56%	38%	
3	46%	38%	62%	
4	11%	3%	0%	
**Charlson Comorbidity Index**	1.97 ± 2.30	1.51 ± 1.70	2.38 ± 1.90	ns
**Prefracture Living**				ns
At home	88%	81%	82%	
Nursing home	8%	19%	9%	
Sheltered housing	4%	0%	9%	
**Prefracture Nursing**				ns
None	48%	29%	9%	
Nursing	52%	71%	82%	
Meals on wheels	0%	0%	9%	
**Time to surgery** <24 h >24 h	100%	100%	100%	ns
**Length of stay** (days)	15.30 ± 5.00	14.80 ± 5.70	13.40 ± 6.80	ns
**One-year mortality** (%)	24%	36%	21%	ns

Data presented as mean ± SD or percentage of each group; ns = not significant.

## Data Availability

The data presented in this study are available on request from the corresponding author. The data are not publicly available due to privacy reasons.
